# Does living alone reshape healthcare use? Longitudinal evidence from older adults in China

**DOI:** 10.1186/s12875-025-03164-7

**Published:** 2026-01-07

**Authors:** Aohan Gao, Yijun Chen, Yue Hou, Mengling Cheng, Jingwen Zhang, Junjie Huang

**Affiliations:** 1 School of Accounting, Tangshan College, No. 9, University West Road, Tangshan, Hebei Province 063000 China; 2 Welsh School of Architecture, Cardiff University, Bute Building, King Edward VII Avenue, Cardiff, CF10 3NB UK; 3 School of Social and Public Administration, East China University of Science and Technology, Meilong Road 130, Shanghai, 200237 China; 4 Department of Sociology, The University of Manchester, Oxford Road, Manchester, M13 9PL England; 5 Faculty of Social Science, University of Stirling, Stirling, Scotland FK9 4LA UK

**Keywords:** Living alone, Healthcare resources utilisation, Older adults, China

## Abstract

**Background:**

China’s rapid population aging and shrinking family structures challenge healthcare access for older adults, especially those living alone. While the link between solitary living and poor health is well-documented, its underlying mechanisms remain unclear. This study examines how living alone shapes healthcare-seeking preferences and behaviors to elucidate its pathways to health in later life.

**Methods:**

Using longitudinal data from the China Family Panel Studies (2010–2020), restricted to a sample of adults aged 60 and above, we employed a linear probability model with individual and time fixed effects to examine the impact of living alone on two key outcomes: the probability of consulting a doctor when ill (*n* = 3,911) and the preference for primary healthcare centers (PHCs) (*n* = 11,956). The model controlled for a set of covariates including demographic characteristics, family attributes, and health status measures. Heterogeneity analyses were conducted by gender, *hukou* status, and household income.

**Results:**

Living alone was associated with a significant reduction in the probability of consulting a doctor (20.2% points), with the effect more pronounced among older women, suggesting that emotional or psychological barriers may exacerbate their vulnerability. In contrast, older adults living alone demonstrated a stronger preference for PHCs (10.9% points), a tendency that was particularly evident among lower-income and urban residents, likely due to the greater accessibility, lower cost, and shorter waiting times of PHCs.

**Implications:**

Strengthening community-based interventions and enhancing the quality and coverage of primary care—particularly in rural areas—are essential to reducing treatment delays and promoting early care-seeking. Policy efforts should prioritise the unique vulnerabilities of older women, low-income individuals, and rural residents by addressing emotional barriers, improving health literacy, and enhancing the accessibility of services. Rebuilding trust in primary care requires sustained investment in provider professionalism, facility infrastructure, and efficient referral systems.

**Supplementary Information:**

The online version contains supplementary material available at 10.1186/s12875-025-03164-7.

## Introduction

### Aging, living alone, and primary care gaps

China’s rapid population aging and the transformation of family structures are intensifying pressures on elderly care and support systems [1, 2]. Driven by rising individualism and urbanization, household size is shrinking, leading to a growing population of older adults living alone. World Health Organization (2024) suggest that by 2040, the proportion of China’s population aged 60 and above will reach 28% [3]. Mirroring this trend, according to 2020 data, the number of households with older adults living alone in China reached 37.29 million, marking a 6.5% increase since 2010. Nationally, 12% of the elderly population lived alone, with the proportion exceeding 15% in provinces such as Anhui [4–6].

To address these challenges, China has invested significantly in its primary healthcare system since the 2009, expanding infrastructure and implementing policies such as drug “zero-markup” and higher insurance reimbursement rates at primary facilities to enhance accessibility and affordability [7]. By 2019, approximately 9,300 community health centers and 26,000 community health stations had been established in urban areas, achieving nearly universal community coverage [8].

Despite these substantial policy efforts, a persistent behavioral gap remains. Multiple studies indicate that many older adults do not significantly increase their likelihood of seeking medical care when experiencing physical discomfort. Among those who do decide to seek care, a considerable proportion bypass primary institutions and opt directly for higher-level hospitals [9–11]. Li et al. (2021) estimate that approximately 40% of older adults in China prefer to seek care at secondary or tertiary hospitals, even though out-of-pocket expenses for services and medications are significantly lower at PHCs [11]. This discrepancy between policy objectives and individual healthcare-seeking behavior underscores the critical need to investigate the underlying preferences and barriers, particularly among vulnerable subgroups like older adults living alone.

### Healthcare utilisation among living alone older individuals

Although extensive research has explored the impact of living alone on various health outcomes, such as mortality risk [12], subjective well-being [13], dementia [14, 15], loneliness [16], mental health [17], and functional independence in daily living [18]. Most studies attribute the negative health effects of living alone to loneliness [13]and lack of social support [17]. However, the mediating pathways through which living alone influences health—particularly the mechanisms operating through healthcare utilization behaviors—remain underexplored. Specifically, living alone may alter individuals’ care-seeking preferences and decision-making processes, thereby affecting their use of primary care and specialized consultation services and ultimately influencing health outcomes. Clarifying this behavioral mechanism is crucial for developing targeted health interventions for older adults living alone.

#### Theoretical framework

The Andersen Model offers a structured framework for examining how living alone influences healthcare utilization among older adults [19]. It categorizes influencing factors into three groups: predisposing characteristics, enabling resources, and need factors [20, 21].

At the level of predisposing characteristics, older adults living alone often experience a lack of social support, particularly daily family interaction. This can lead to delayed symptom recognition and reduced initial willingness to seek medical care [22, 23]. Identity theory offers a deeper explanation for this phenomenon: the absence of social or family relationships deprives individuals of an important source of social affirmation and self-worth, which may exacerbate psychological vulnerability and foster fear and avoidance in healthcare decision-making [24, 25]. This psychologically driven mechanism, rooted in identity change, may be particularly pronounced among older women, who have traditionally served as family caregivers [26].

Regarding enabling resources, older adults living alone often face greater structural barriers in accessing healthcare. These include transportation difficulties, challenges in scheduling appointments, problems comprehending medical information, as well as financial constraints and the digital divide [27, 28]. These obstacles are further amplified by urban-rural disparities reinforced by the hukou system, resulting in more disadvantageous circumstances for older adults living alone in rural areas [29–32].

Finally, in terms of need factors, living alone may create a mismatch between subjectively perceived health needs and objectively evaluated medical needs. Without daily family observation and reminders, older adults living alone may fail to detect health changes in a timely manner or underestimate the severity of their symptoms, thereby delaying necessary medical intervention.

#### Empirical evident

Academic consensus regarding the care-seeking behaviors of solo-dwelling elders remains fragmented, particularly within China’s unique filial piety culture and transitioning healthcare structure. While some international evidence suggests heightened clinical engagement due to diminished informal care resources [33], contrasting findings [34] demonstrates systemic disadvantages in care accessibility among Chinese empty-nest elders. This epistemological conflict necessitates rigorous examination of the underlying mechanisms governing medical resource allocation in solitary aging contexts.

Within the Chinese context, substantial empirical evidence reveals the multidimensional health vulnerabilities faced by older adults living alone, which form the physiological and psychological basis for their distinct healthcare needs and utilization patterns. Utilizing longitudinal data from the Chinese Longitudinal Healthy Longevity Survey (CLHLS), the empirical study confirmess that while older adults living alone show no significant differences in objective physiological indicators compared to their non-solitary counterparts, they exhibit extremely high vulnerability in the mental health dimension, with depressive symptoms being particularly prominent [35]. This finding resonates with the survey in Qiqihar City, which indicated that the mental health service needs of older adults living alone—such as psychological communication, knowledge training, counseling, and monitoring—are comprehensively higher than those of older adults living with families [36]. These needs are most acute among economically disadvantaged older women with poor physical health and limited self-care ability. The comparative study further corroborates that older adults living alone report significantly higher levels of negative affect and negative experiences [37].

This multidimensional vulnerability, particularly the psychological burden, significantly reshapes the pathways and efficiency of healthcare utilization among older adults living alone through specific mechanisms. Firstly, regarding the psychological-behavioral pathway, negative emotions such as loneliness and depression not only directly undermine their motivation for health management but are also likely to lead to underutilization of preventive care [37]. When health crises occur, they are consequently forced to rely more heavily on emergency services and post-acute care [38]. Secondly, the breakdown of social support networks constitutes a key barrier. A case study reveals that rural older women living alone experience significant delays in seeking medical care due to the absence of companionship and reminders [39]. This “support vacuum” shifts tasks that could be handled by families—such as daily health monitoring and accompanying medical visits—onto the formal healthcare system, thereby increasing its burden. Furthermore, The interplay of economic deprivation and institutional gaps compounds health disparities. As evidenced in multiregional studies, older adults living alone disproportionately experience financial constraints that transform healthcare navigation into economically-mediated decisions [40, 41]. Such fiscal pressures institutionalize defensive healthcare consumption patterns—prioritizing immediate needs while deprioritizing preventive and non-urgent care, ultimately cementing their dependence on state-sponsored safety nets.

It is crucial to assess whether older adults living alone in China, a rapidly growing demographic facing declining health, reduced social connections, and limited family support—can effectively and appropriately access healthcare resources. This study, based on the Chinese national dataset, China Family Panel Studies (CFPS), Two-Way Fixed Effect model to estimate the causal relationship between living alone and healthcare utilization preferences, aiming to answer three key questions:

1. Compared to older individuals living with family members, are those living alone more likely to seek medical care when experiencing physical discomfort?
2. Compared to older individuals living with family members, do older individuals living alone prefer primary healthcare services more strongly when seeking medical care?
3. To what extent do factors such as gender, household registration status, and household income mediate this relationship?

The structure of this paper is as follows: Method section reviews the data sources and variables; Results section presents the model specifications; Discussions section presents the empirical results and explores the potential heterogeneous effects of living alone on older individuals’ preferences for healthcare resource utilisation; the final section provides a discussion and conclusion.

## Method

### Data

This study utilises secondary data from the China Family Panel Studies (CFPS), a nationally representative biennial longitudinal survey initiated in 2010 by the Institute of Social Science Survey (ISSS) at Peking University. The CFPS collects extensive data on social, economic, demographic, educational, and health-related aspects of Chinese households, communities, and individuals. Comprehensive methodological details, questionnaires, and dataset documentation have been published previously and are accessible through the CFPS official website. Additional information can be found in Xie and Hu (2014) [42].

**Fig. 1:**
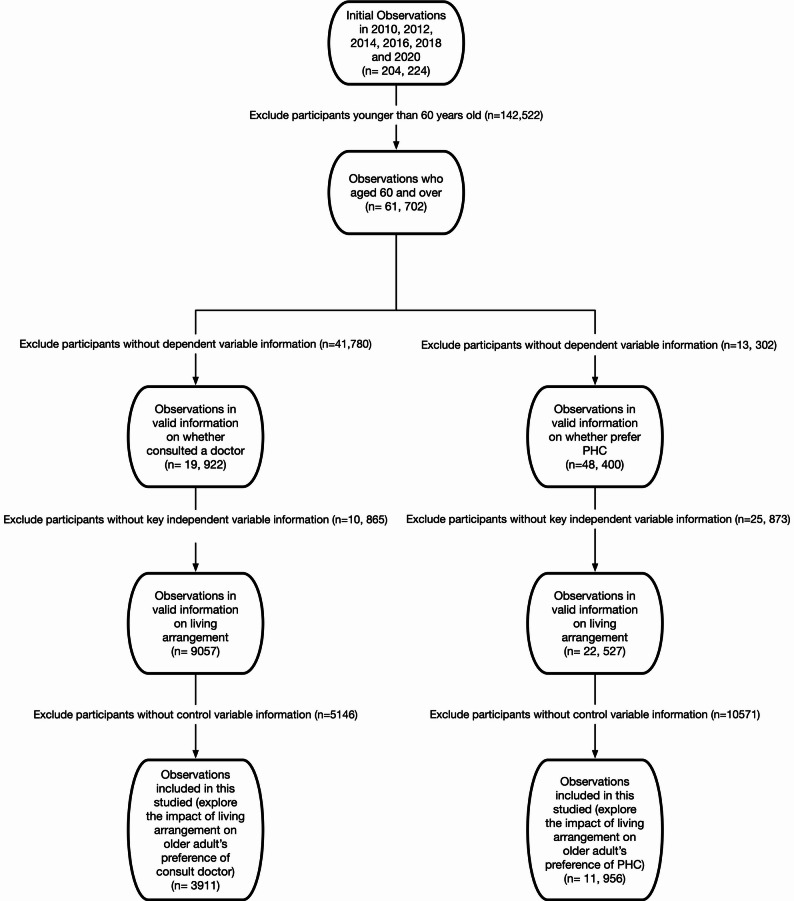
Data Structure

For this analysis, data from six waves (2010, 2012, 2014, 2016, 2018, and 2020) were pooled, focusing on respondents aged 60 and above. We applied a series of exclusion criteria to the pool dataset. First, individuals younger than 60 in the survey year were excluded (*n* = 142,522). Additionally, participants with missing data on regression variables were removed.^1^ Due to data limitations, the two aspects of healthcare utilisation analysed in this study are based on partially overlapping samples. In the CFPS, data on doctor consultation preferences are drawn only from respondents who reported feeling unwell in the past two weeks, as only they were asked whether they sought medical care. Consequently, the sample for analysing doctor consultations is smaller and not representative of all older adults. However, since recent illness can be considered randomly distributed, the risk of sample selection bias is minimal. The final analytical sample includes 3,911 observations for doctor consultation and 11,956 for preference for primary healthcare centres. More detailed information is presented in Fig. 1.

### Variables

#### Outcome variables

##### Outcome variable A: consulted doctors

The outcome variable, *Doctor Consultation*, was derived from two CFPS survey questions. Respondents were first asked whether they had experienced physical discomfort in the past two weeks. Those who answered “yes” were then asked if they sought medical consultation. The variable was coded as 1 for respondents who sought care and 0 for those who did not.

##### Outcome variable B: primary health care (PHC) preference

The second outcome variable, *PHC Preference*, reflects respondents’ usual choice of healthcare provider. They were asked where they typically seek care, with options classified as primary healthcare (PHC) or non-PHC institutions. The variable was coded as 1 for those choosing PHC institutions and 0 for non-PHC options.

#### Key explanatory variables

The key independent variable is “Living Alone”, derived from the survey question “Who do you live with?” Respondents who reported “living alone” were assigned a value of 1 for the binary variable “Living Alone”. Conversely, respondents who indicated living with “children, spouse, grandchildren, or others” were assigned a value of 0.

#### Other explanatory variables

The Andersen model of health service utilisation identifies three primary categories of factors influencing health service use: predisposing, enabling, and need-based factors [43]. This study examines medical treatment preferences and hypothesises that individual preferences significantly impact health service utilisation as shaped by these three factors. Hence, this study detailed the specific time-varying explanatory variables used in the analysis and provided a rationale for their inclusion.

This study considers age, age square and marital status as predisposing factors. The authors consider household income and basic social medical insurance as enabling factors. We consider chronic disease status and intensity of physical discomfort as need-based factors. The detailed variable definitions are presented in Supplementary Table S1.

#### Heterogeneous variables

To further investigate the impact of living alone on healthcare utilisation preferences among older adults, this study explores the heterogeneity of this relationship across three key moderating variables.

- Gender: Gender differences influence health perceptions and healthcare-seeking behaviours. Gender is coded as a binary variable: 0 = female, 1 = male.
- Household Registration Type (Hukou): Hukou status is a key determinant of socioeconomic standing and eligibility for public services, including healthcare. We derived this variable directly from the survey question regarding respondents’ registered household type. It was operationalized as a binary measure: 0 = rural hukou, 1 = urban hukou.
- Household Income: As a key indicator of financial capacity, household income shapes healthcare-seeking behaviour. For the heterogeneity analysis, household income was dichotomized based on the sample mean: 1 = above-average income, 0 = at or below-average income.

### Analytical method

The research sample in this study is drawn from six rounds of surveys conducted across 25 provinces, autonomous regions, and municipalities in China. Given that data from different time periods and regions do not satisfy the homogeneity assumption regarding technological, environmental, and social factors, this study employs a fixed effects regression model. All data management and statistical analyses were performed using Stata, Version 15. This included the processes of data cleaning, variable creation, and sample construction. The estimation of our primary linear probability models with fixed effects, alongside the supplementary logistic models and heterogeneity analyses, was carried out using Stata’s built-in commands.

However, Timoneda (2021) highlights that maximum likelihood estimation in fixed effects models can lead to substantial sample loss, as groups with no variation in the dependent variable are dropped [44]. Additionally, coefficient estimates become increasingly unreliable as more covariates are included. When estimating fixed effects with a binary outcome variable, the Linear Probability Model with Fixed Effects yields more accurate estimates and predicted probabilities than maximum likelihood estimation, particularly when the dependent variable contains fewer than 25%.

In this study, the probability of consulting a doctor is 81.69%, while the probability of not consulting a doctor is 18.31%, meeting Timoneda (2021) criterion for rare events [44]. For another binary dependent variable—primary healthcare utilisation (PHC)—the probability of occurrence is 64.81%, with non-occurrence at 35.19%. Timoneda (2021) suggests that when the proportion of ones (or zeros) falls between 25% and 50%, MLE performs only marginally better than LPMFE, primarily because nonlinear models improve accuracy at extreme values [44]. However, as this study focuses on the average causal effect of living alone on preferences for healthcare utilisation, the marginal accuracy gains of nonlinear models at extreme values, and achieved at the cost of reduced sample size are not the primary concern.

Given this context, the LPMFE is expected to provide more robust estimates than Logit and its extensions, which rely on MLE. Therefore, this study adopts the LPMFE as the benchmark model.

The benchmark regression model is set as follows:

1 $$\begin{aligned} {\mathrm{MTP}}_{\mathrm{it}}=&{\mathrm\alpha}_0+{\mathrm\alpha}_1\;{\mathrm C}_{\mathrm{it}}+\\&{\mathrm\alpha}_2\;{\mathrm P}_{\mathrm{it}}+{\mathrm\alpha}_3\;{\mathrm E}_{\mathrm{it}}+\\&{\mathrm\alpha}_4\;{\mathrm N}_{\mathrm{it}}+{\mathrm T}_{\mathrm t}+{\mathrm M}_{\mathrm i}+{\mathrm\mu}_{\mathrm{it}} \end{aligned}$$

In Eq. (1), MTP_it_ is the healthcare utilisation preference (including whether consulted doctors and primary health care preference) of individual i (i = 1,…, N) at time t. C_it_ is the critical, independent variable of whether the respondent lives alone. The healthcare utilisation preference also regresses on predisposing factors (P_it_), enabling factors (E_it_) and needs-based factors (N_it_). M_i_ represents the individual-fixed effects to control for all time-invariant heterogeneity (e.g., gender, educational background, and Hukou status). Time-fixed effects ($\:{T}_{t}$) are included to account for common temporal shocks, such as policy reforms or the COVID-19 pandemic. Our identification strategy relies on within-individual variation over time, estimating how a change in living arrangements is associated with a change in healthcare utilization preferences. µ_it_ is the random error term.

## Results

### Sample distribution

The study investigates the effect of living alone on healthcare utilisation behaviours among older adults, specifically examining two outcomes:

1. the probability of consulting a doctor when unwell and.
2. preferences for primary health centres over higher-level facilities.

Regarding (1), consulting a doctor was measured based on respondents who reported illness within the two weeks preceding the survey. Of 61,702 initial observations, 19,922 respondents (32.29%) provided valid data for this variable, with 15,201 reporting they sought medical consultation and 4,721 not seeking care. The short recall period mitigates potential selection bias despite the reduced sample size.

Regarding (2), preference for primary healthcare centres (PHCs) was derived from a broader sample. Among 61,702 observations, 48,400 respondents (78.44%) reported valid preferences, with 31,076 favouring primary health centres and 17,324 preferring higher-level facilities, such as hospitals.

The key explanatory variable, living arrangement, was defined as whether the respondent lived alone. Among the initial sample, 27,365 respondents (44.35%) provided valid information, with 4,576 living alone and 22,789 cohabiting with family.

After applying these criteria, the final analytical sample for this study consisted of 3911 observations to explore the impact of living alone on consulting a doctor and 11,956 observations to explore the impact of living alone on preference for primary healthcare centres. The sample distribution is summarised in Table 1.^2^ The descriptive analyses for benchmark regression were conducted using STATA version 15.0 software, with a significance level of *P* < 0.05.

**Table 1:** Descriptive statistics

Variables	Whether consulted a doctor							
	All observations			Not living alone		Living alone		MeanDiff
	*N*	Mean	sd	*N*	Mean	*N*	Mean	
Age	3911	71.35	9.97	3035	70.63	876	73.85	-3.22***
Household income	3911	34554.93	45287.53	3035	39140.29	876	18668.45	20471.85***
Household size	3911	4.72	2.23	3035	5.52	876	1.92	3.60^***^
	Freq.	Percent	Cum.	Freq.	Percent	Freq.	Percent	
Whether consulted a doctor								
No	716	18.31	18.31	556	18.32	160	18.26	0.00
Yes	3,195	81.69	100	2,479	81.68	716	81.74	
Living alone								
No	3,035	77.6	77.6					
Yes	876	22.4	100					
Marital status								
Single	113	3.00	2.89	12	0.03	101	11.53	-2.23***
Married (with spouse)	2,887	73.82	76.71	2,823	93.0	64	7.31	
Cohabiting	54	1.38	78.09	53	1.7	1	0.11	
Divorced	86	2.20	80.29	9	0.30	77	8.79	
Widowed	771	19.71	100	138	4.55	633	72.26	
Chronic illness								
No	2,202	56.3	56.3	1,710	56.34	492	56.16	0.00
Yes	1,709	43.7	100	1,325	43.66	384	43.84	
Type of insurance								
None insurance	321	8.21	8.21	232	7.64	89	10.16	0.17***
Public medical care	120	3.07	11.28	83	2.73	37	4.22	
Urban employee medical insurance	386	9.87	21.15	299	9.85	87	9.93	
Urban resident medical insurance	291	7.44	28.59	209	6.89	82	9.36	
New rural cooperative medical care	2,793	71	100	2,212	72.88	581	66.32	
Intensity of physical discomfort								
Not serious	534	13.65	13.65	430	14.17	104	11.87	-0.05
Moderate	1,404	35.9	49.55	1,077	35.49	327	37.33	
Serious	1,973	50.45	100	1,528	50.35	445	50.8	
Gender								
Female	1861	57.19	57.19	1,367	55.14	494	63.74	0.09***
Male	1393	42.81	100.00	1112	44.86	281	36.26	
Hukou								
Rural	2882	77.81	77.81	2250	80.82	632	73.57	-0.28^***^
Urban	822	22.19	100	605	19.18	227	26.43	
Independent outdoor activity								
No	407	11.06	11.06	309	10.82	98	11.88	0.01
Yes	3,273	88.94	100.00	2,546	89.18	727	88.12	
Total	3,911	100		3,035	100	876	100	

Variables	Primary Health Care							
	All observations			Not living alone		Living alone		MeanDiff
	N	Mean	sd	N	Mean	N	Mean	
Age	11,956	71.34	10.12	9775	70.76	2181	73.93	-3.17***
Household income	11,956	40121.52	50144.35	9775	44672.23	2181	19725.74	24946.49***
Household size	11,956	4.83	2.21	9775	5.49	2181.00	1.89	2.4^***^
	Freq.	Percent	Cum.	Freq.	Percent	Freq.	Percent	
PHC								
No	4,207	35.19	35.19	3,471	35.51	736	33.75	-2.00
Yes	7,749	64.81	100	6,304	64.49	1,445	66.25	
Living alone								
No	9,775	81.76	81.76					
Yes	2,181	18.24	100					
Marital status								
Single	300	2.51	2.51	68	0.07	232	10.64	-2.18***
Married (with spouse)	9,466	79.17	81.68	9,242	94.55	224	10.27	
Cohabiting	19	0.16	81.84	15	0.15	4	0.18	
Divorced	293	2.45	84.29	121	0.01	172	7.89	
Widowed	1,878	15.71	100	329	0.07	1,549	71.02	
Chronic illness								
No	8,546	71.48	71.48	7,066	72.29	1,480	67.86	-0.04***
Yes	3,410	28.52	100	2,709	27.71	701	32.14	
Type of insurance								
None insurance	955	7.99	7.99	740	7.57	215	9.86	0.06**
Public medical care	430	3.6	11.58	353	3.61	77	3.53	
Urban employee medical insurance	1,451	12.14	23.72	1,204	12.32	247	11.33	
Urban resident medical insurance	1,044	8.73	32.45	867	8.87	177	8.12	
New rural cooperative medical care	8,076	67.55	100	6,611	67.63	1,465	67.17	
Gender								
Female	4776	48.82	48.82	3728	47.19	1048	55.66	0.09^***^
Male	5007	51.18	100	4,172	52.81	835	44.34	
Hukou								
Rural	8,258	73.05	73.05	6701	72.88	1577	74.04	-0.02
Urban	3047	26.95	100	2494	27.12	553	25.96	
Independent outdoor activity								
No	725	6.39	6.39	544	5.85	181	8.80	0.03^***^
Yes	10,615	93.61	100.00	8740	94.14	1875	91.20	
Total	11,956	100		9775	100	2181	100	

A slight reduction in observations occurred in the robustness checks and heterogeneity analysis due to missing values in key variables. In the “Whether consulted a doctor” panel (baseline *N* = 3,911), complete cases numbered 3,254 for gender (-16.8%), 3,704 for hukou (-5.3%), and 3,680 for independent outdoor activity (-5.9%). Similarly, in the “Primary Health Care” panel (baseline *N* = 11,956), complete cases numbered 9,783 for gender (-18.2%), 11,305 for hukou (-5.4%), and 11,304 for independent outdoor activity (-5.5%)

** *p* < 0.05, ***p < 0.01

### Benchmark model estimation results

This study examines the impact of living alone on healthcare utilisation preferences among older adults in China, focusing on two key dimensions: (1) the probability of consulting a doctor when experienced physical discomfort, and (2) the preference for primary healthcare centers (PHCs) over other levels of medical facilities.

Table 2 presents the regression results for both outcomes. To ensure robustness of the method, we estimated models with different levels of fixed effects. Columns 1 and 3 include only individual fixed effects, while columns 2 and 4 incorporate both individual and time fixed effects, with the latter serving as our benchmark specification. The results reveal a significant negative association between living alone and the propensity to seek medical consultation. Specifically, older adults living alone exhibit a 18.5 to 20.2%-point reduction in the probability of consulting a doctor (columns 1 and 2, Table 2). In contrast, living alone is associated with an 8.4 to 10.9%-point increase in the probability of utilising primary healthcare centers (columns 3 and 4, Table 2). It is worth noting, however, that the positive relationship between living alone and PHC utilisation loses statistical significance in specifications that do not control for time fixed effects (column 3, Table 2). This suggests that the observed preference may be sensitive to unobserved time-varying factors shared by all individuals in a given year, such as shifts in national healthcare policy, macroeconomic conditions, or public health campaigns.

**Table 2:** TWFE estimates results on the probability of consulting Doctors and going to PHC

	Consulted a doctor		Primary Health Care	
	(1)	(2)	(3)	(4)
Living alone	-0.185^**^	-0.202^**^	0.084	0.109^*^
	(0.080)	(0.081)	(0.054)	(0.054)
Age	-0.010	-0.012	-0.016^*^	-0.006
	(0.010)	(0.012)	(0.008)	(0.011)
Age square	0.000	0.000	0.000^**^	0.000
	(0.000)	(0.000)	(0.000)	(0.000)
Married	-0.201	-0.190	0.195^*^	0.213^*^
	(0.322)	(0.325)	(0.110)	(0.107)
Cohabiting	-0.162	-0.139	0.284^*^	0.333^**^
	(0.393)	(0.392)	(0.152)	(0.146)
Divorced	0.199	0.205	0.056	0.109
	(0.220)	(0.240)	(0.091)	(0.089)
Widowed	0.006	0.002	0.009	0.094
	(0.298)	(0.304)	(0.118)	(0.125)
Chronic illness	0.076^***^	0.068^***^	-0.068^***^	-0.062^***^
	(0.014)	(0.014)	(0.013)	(0.013)
Household income	-0.009	0.011	0.005	-0.002
	(0.007)	(0.008)	(0.004)	(0.007)
Public medical care	0.118	0.113	-0.082	-0.090^*^
Urban employee medical insurance	(0.077)	(0.074)	(0.053)	(0.050)
Urban resident medical insurance	0.042	0.020	-0.073^*^	-0.066^*^
New rural cooperative medical care	(0.062)	(0.063)	(0.039)	(0.038)
Public medical care	0.092^*^	0.079	-0.045	-0.036
Urban employee medical insurance	(0.046)	(0.049)	(0.031)	(0.030)
Urban resident medical insurance	0.039	0.030	0.019	0.021
	(0.028)	(0.031)	(0.026)	(0.028)
Moderate physical discomfort	0.151^***^	0.148^***^		
	(0.038)	(0.037)		
Serious physical discomfort	0.236^***^	0.235^***^		
	(0.038)	(0.038)		
Constant	1.340^**^	1.157	1.028^***^	0.742
	(0.621)	(0.739)	(0.301)	(0.476)
Time Fixed Effects	No	Yes	No	Yes
Individual Fixed Effects	Yes	Yes	Yes	Yes
Number of observations	3911	3911	11,956	11,956
Adjusted R2	0.549	0.556	0.577	0.588

To test the robustness of the model, we explored fixed effects at different levels. In the first column, only employment-time fixed effects are included. The second column incorporates both employment-time and individual fixed effects (benchmark model). Similarly, the third column includes employment-time fixed effects, the fourth column combines employment-time and individual fixed effects (benchmark model)

Standard errors in parentheses, * *p* < 0.1, ** *p* < 0.05, *** *p* < 0.01

Results for control variables align with theoretical expectations. Health status significantly influences healthcare-seeking behaviour: chronic illness increases the likelihood of consulting physicians. Still, it reduces the preference for primary healthcare centres, reflecting a greater need for specialized care typically available at higher-tier facilities. Similarly, individuals reporting moderate or severe physical discomfort are more likely to seek medical attention, consistent with expectations. Significant heterogeneity is also observed by insurance type. Older adults covered by the Urban Resident Basic Medical Insurance (URBMI) strongly prefer specialized and general hospitals over primary care centres. However, URBMI coverage does not significantly affect the decision to seek care when ill. Differences in benefit design, reimbursement rates, and perceived quality of care may explain this pattern. URBMI generally offers more favourable reimbursement for hospital-based services (49,50), and higher-tier hospitals are often viewed as offering superior expertise and diagnostic capacity, encouraging beneficiaries to bypass primary care.

To further assess the robustness of our main findings, we conducted a series of supplementary analyses, as reported in Appendix Tables S3.1 and S3.2. These tests included: estimating nonlinear logistic models (columns 1–2), controlling for the ability to go outside independently (column 3), adjusting for household size (column 4), clustering standard errors at the individual level (column 5), and restricting the sample to adults aged 65 and older (column 6). Across nearly all specifications, the results consistently support our primary conclusions: older adults living alone are less likely to consult a doctor when experiencing physical discomfort, yet more inclined to use primary healthcare centers compared to those living with others.

### Heterogeneity analysis

The benchmark regression analysis reveals a significant influence of living alone on healthcare utilisation preferences among older adults, encompassing the decision to seek medical care and the selection of healthcare facility level. To further explore potential heterogeneity in healthcare preferences, subsequent analyses examine whether these preferences vary across different demographic and socioeconomic groups. Specifically, the investigation focuses on three key dimensions: gender, household registration status (hukou), and household income.

#### Gender

Table 3 presents the gender-specific analysis of how living alone influence healthcare utilisation preferences among older adults. Columns (1) and (2) display the probability of consulting a doctor when feeling unwell for females and males, respectively, while Columns (3) and (4) report the probability of choosing primary healthcare services.

**Table 3:** Heterogeneity analysis: gender

	Consulted a doctor		Primary Health Care	
	Female	Male	Female	Male
	(1)	(2)	(3)	(4)
Living alone	-0.206^**^	-0.151	0.023	0.131
	(0.092)	(0.145)	(0.079)	(0.099)
Age	-0.023	-0.011	-0.010	0.008
	(0.014)	(0.023)	(0.013)	(0.017)
Age square	0.000	0.000	0.000	-0.000
	(0.000)	(0.000)	(0.000)	(0.000)
Married	-0.358^**^	-0.173	0.317^*^	-0.017
	(0.140)	(0.577)	(0.174)	(0.138)
Cohabiting	0.051	-0.355	0.722^***^	0.102
	(0.403)	(0.603)	(0.179)	(0.227)
Divorced	0.605^***^	0.075	0.249	-0.093
	(0.175)	(0.606)	(0.207)	(0.146)
Widowed	-0.075	-0.119	0.235	-0.087
	(0.053)	(0.680)	(0.160)	(0.173)
Chronic illness	0.047^**^	0.100^***^	-0.042^**^	-0.074^***^
	(0.023)	(0.028)	(0.016)	(0.021)
Household income	0.006	0.011	-0.006	0.004
	(0.010)	(0.016)	(0.006)	(0.009)
Public medical care	0.053	0.093	-0.069	-0.057
Urban employee medical insurance	(0.167)	(0.132)	(0.076)	(0.075)
Urban resident medical insurance	-0.010	-0.062	-0.092^**^	-0.035
New rural cooperative medical care	(0.115)	(0.108)	(0.040)	(0.087)
Public medical care	0.108	-0.039	-0.048	-0.053
Urban employee medical insurance	(0.100)	(0.107)	(0.057)	(0.045)
Urban resident medical insurance	0.072	-0.093	0.028	0.051
	(0.044)	(0.094)	(0.028)	(0.051)
Moderate physical discomfort	0.141^***^	0.143^**^		
	(0.040)	(0.063)		
Serious physical discomfort	0.218^***^	0.240^***^		
	(0.044)	(0.065)		
Constant	1.862^**^	1.259	0.851	0.329
	(0.675)	(1.229)	(0.556)	(0.749)
Number of observations	1861	1393	4776	5077
Adjusted R2	0.558	0.561	0.615	0.603

Columns (1)-(4) all employment-time and individual fixed effects

* *p* < 0.1, ** *p* < 0.05, *** *p* < 0.01

The results show that gender heterogeneity appears primarily in the decision to seek medical care, not in the choice of healthcare provider. This finding aligns with gender role theory [26], which posits that women are more closely tied to family roles and responsibilities. As a result, the loss of family connections may have a stronger psychological and behavioural impact on women, leading to greater emotional distress, social withdrawal, and hesitation in seeking medical care. This may partly explain why older women living alone often report worse physical and mental health than their male counterparts [45, 46].

#### Household registration type - hukou (urban vs. rural)

Table 4 presents the results of the heterogeneity analysis by household registration type (urban vs. rural), assessing the impact of living alone. Columns (1) and (2) display the probability of consulting a doctor for rural and urban residents, respectively, while Columns (3) and (4) report the probability of selecting primary healthcare services. The findings reveal notable differences in healthcare utilisation patterns between rural and urban populations.

**Table 4:** Heterogeneity analysis: household registration Type - Hukou

	Consulted doctors		Primary Health Centres	
	Rural	Urban	Rural	Urban
	(1)	(2)	(3)	(4)
Living alone	-0.157^**^	-0.236^*^	0.068	0.345^***^
	(0.075)	(0.120)	(0.066)	(0.083)
Age	-0.016	0.006	-0.001	-0.009
	(0.013)	(0.044)	(0.013)	(0.018)
Age square	0.000	0.000	0.000	0.000
	(0.000)	(0.000)	(0.000)	(0.000)
Married	-0.260^***^	-0.005	0.206	0.070
	(0.078)	(0.896)	(0.147)	(0.058)
Cohabiting	-0.085	0.011	0.436^**^	-0.207
	(0.276)	(0.096)	(0.183)	(0.219)
Divorced	0.085^*^	0.699	0.149	-0.156
	(0.045)	(0.693)	(0.104)	(0.145)
Widowed	-0.154	0.434	0.127	-0.214
	(0.113)	(0.839)	(0.170)	(0.128)
Chronic illness	0.068^***^	0.071^*^	-0.066^***^	-0.038^**^
	(0.020)	(0.039)	(0.018)	(0.017)
Household income	0.008	0.008	-0.000	0.001
	(0.010)	(0.032)	(0.004)	(0.021)
Public medical care	-0.120	0.156^*^	-0.085	-0.077
Urban employee medical insurance	(0.162)	(0.088)	(0.115)	(0.053)
Urban resident medical insurance	-0.035	0.041	-0.101	-0.070^*^
New rural cooperative medical care	(0.265)	(0.056)	(0.133)	(0.036)
Public medical care	0.175	0.129^*^	0.017	-0.056
Urban employee medical insurance	(0.151)	(0.064)	(0.096)	(0.034)
Urban resident medical insurance	0.012	0.150^**^	0.016	0.046
	(0.040)	(0.068)	(0.026)	(0.053)
Moderate	0.125^***^	0.229^**^		
	(0.029)	(0.107)		
Serious	0.209^***^	0.303^***^		
	(0.035)	(0.082)		
Constant	1.527^**^	-0.122	0.632	0.698
	(0.617)	(2.730)	(0.571)	(0.778)
Number of observations	2882	822	8258	3047
Adjusted R2	0.560	0.600	0.531	0.589

Standard errors in parentheses

Columns (1)-(4) all employment-time and individual fixed effects

* *p* < 0.1, ** *p* < 0.05, *** *p* < 0.01

Living alone reduces the likelihood of consulting a doctor, regardless of their registration type. This could stem from factors such as limited availability of healthcare resources, lower health literacy, or logistical challenges, including transportation difficulties.

The analysis of primary healthcare preferences reveals a notable contrast between rural and urban residents. Among rural residents, living alone has a small positive effect (0.068) on the probability of choosing primary healthcare services, though this effect is statistically insignificant. In contrast, for urban residents, living alone is associated with a substantial and statistically significant increase of 34.5% points in primary healthcare utilisation.

This disparity likely stems from fundamental differences in the accessibility and quality of primary healthcare between rural and urban China. Rural facilities face structural challenges such as low wages, limited benefits, high staff turnover, and an ageing workforce, reducing professionalism and lower trust in local services [47]. Additionally, weak public health infrastructure—particularly in digital systems—hampers care coordination. The absence of efficient electronic records and referral mechanisms limits patient transfers and reduces service efficiency, discouraging rural residents from using primary care and hindering access to timely, effective treatment [47].

#### Household income

Table 5 presents the results of the heterogeneity analysis by household income, assessing the impact of living alone. Columns (1) and (2) report the probability of consulting a doctor for below- and above- average income households, respectively, while Columns (3) and (4) examine the probability of selecting primary healthcare services.

**Table 5:** Heterogeneity analysis: household income

	Consulted doctors		Primary Health Centres	
	below- average	above- average	below- average	above- average
	(1)	(2)	(3)	(4)
Living alone	-0.169^*^	-0.180^***^	0.140^**^	0.131
	(0.086)	(0.059)	(0.067)	(0.248)
Age	-0.016	0.017	-0.015	-0.000
	(0.014)	(0.034)	(0.010)	(0.013)
Age square	0.000	-0.000	0.000	0.000
	(0.000)	(0.000)	(0.000)	(0.000)
Married	0.145	-1.271^***^	0.284^***^	0.135
	(0.347)	(0.139)	(0.083)	(0.137)
Cohabiting	0.398	-1.080^***^	0.596^***^	0.130
	(0.541)	(0.218)	(0.135)	(0.144)
Divorced	0.351	-0.150	0.169^*^	-0.049
	(0.312)	(0.129)	(0.085)	(0.134)
Widowed	0.211	-1.153^***^	0.146	0.102
	(0.364)	(0.184)	(0.110)	(0.227)
Chronic illness	0.070^***^	0.127^***^	-0.057^***^	-0.054
	(0.025)	(0.041)	(0.019)	(0.032)
Household income	0.006	-0.032	0.000	-0.000
	(0.013)	(0.038)	(0.006)	(0.017)
Public medical care	-0.138	0.310^**^	-0.135^**^	-0.015
Urban employee medical insurance	(0.105)	(0.113)	(0.062)	(0.061)
Urban resident medical insurance	0.012	0.249^**^	-0.112	-0.019
New rural cooperative medical care	(0.105)	(0.099)	(0.071)	(0.039)
Public medical care	0.030	0.276^***^	-0.152^**^	0.038
Urban employee medical insurance	(0.051)	(0.096)	(0.057)	(0.048)
Urban resident medical insurance	-0.009	0.294^**^	-0.008	0.058
	(0.047)	(0.108)	(0.029)	(0.047)
Moderate physical discomfort	0.100^***^	0.167^*^		
	(0.032)	(0.085)		
Serious physical discomfort	0.198^***^	0.171^*^		
	(0.046)	(0.088)		
Constant	1.305	0.944	1.134^**^	0.360
	(0.785)	(1.600)	(0.440)	(0.618)
Number of observations	2201	684	7265	2500
Adjusted R2	0.562	0.600	0.607	0.651

Standard errors in parentheses

Columns (1)-(4) all employment-time and individual fixed effects

* *p* < 0.1, ** *p* < 0.05, *** *p* < 0.01

Living alone reduce the probability of consulting a doctor for low- and high-income-income living alone older adults. Meanwhile, the permutation test shows that these two adverse effects have no statistically significant difference.

The analysis reveals a clear income-based contrast in primary healthcare preferences. Living alone increases the likelihood of using primary care by 14% points among lower-income households, suggesting greater reliance on its affordability and accessibility. In higher-income households, although the effect is slightly larger (0.131), it is not statistically significant, likely because greater financial resources allow access to a wider range of healthcare options, including specialised services.

## Discussions

This study utilizes longitudinal data from the China Family Panel Studies (CFPS) to systematically analyze the impact of living alone on healthcare utilization among older adults in China. The findings reveal a noteworthy dual pattern: while living alone significantly reduces the probability of seeking medical care, it simultaneously increases the preference for primary healthcare institutions (PHCs) among those who do seek care. While this pattern resonates with Yan and Li’s (2022) observations regarding the health vulnerability of solitary elders [35], our study advances the discourse by leveraging fixed-effects models to disentangle the influence of time-invariant individual traits—such as inherent personality or chronic health conditions—from the structural consequences of living alone. This methodological refinement shifts the explanatory focus from individual predispositions to the institutional and social voids created by solitary living.

### Structural vacuum and behavioral withdrawal

A central finding of this study is that living alone reduces the probability of seeking medical care by 20.2%. While studies often attribute such behavioral withdrawal to personality traits like social withdrawal or depressive tendencies [16, 17, 35, 36], our longitudinal fixed-effects models control for all time-invariant individual heterogeneity. This allows us to argue that the observed decline in healthcare utilization stems not from intrinsic character but from the structural vacuum left by the absence of a co-resident family.

In family health sociology, family members constitute an informal lay referral system: they monitor symptoms, validate health concerns, and provide critical “cues to action.” Once an elder lives alone, this system collapses. Medical decisions—from recognizing symptoms to arranging a hospital visit—become fully individualized. Without external validation or encouragement, health issues are more easily downplayed or deferred. Moreover, the logistical and cognitive costs of navigating the healthcare system—such as appointment scheduling, travel, and in-hospital navigation—become fully internalized. What was once a shared burden among family members now falls entirely on the solitary elder. Thus, the observed decline in care-seeking might not a reflection of “stubbornness” or “independence,” but a rational behavioral retreat in the face of elevated physical and psychological transaction costs.

### Constrained adaptation and defensive healthcare consumption

While the preference for PHCs among elders living alone could be viewed as support for primary care, it is important to consider alternative explanations. Grounded in Andersen’s behavioral model, we posit that this pattern may better reflect a constrained adaptation to the loss of key enabling resources, such as the organizational support of a family.

In China’s healthcare landscape, tertiary hospitals are characterized by complex, often digitized processes and high crowding. For elders with family support, adult children often serve as “navigators,” making advanced care accessible. Solitary elders, by contrast, face what we term downgraded substitution: they opt for PHCs not out of preference, but as a defensive healthcare consumption strategy. PHCs offer geographical proximity, simpler procedures, and familiar environments—attributes that align with their diminished capacity to cope with systemic complexity. This represents a survival strategy aimed at accessing the most feasible care within their constrained resources, rather than an expression of genuine choice.

### Heterogeneity analysis: the non-substitutability of social support and the loss of gender role identity

Heterogeneity analysis in this study reveals deeper mechanisms that extend beyond economic factors. One notable finding is the consistent negative effect of living alone on healthcare-seeking behavior across income groups, which might suggest that social support may act as a non-substitutable resource: even among older adults with higher incomes, the absence of a “health governance agent”—someone who monitors symptoms, facilitates navigation, and encourages treatment—cannot be fully compensated by monetary means. This underscores the need for policy interventions that address social support systems, in addition to economic measures.

Furthermore, the more pronounced discouraging effect of living alone on healthcare-seeking among older women can be further understood through the lens of identity theory [24]. In traditional Chinese families, older women often occupy the central role identity of “caregiver” and family health manager [23, 39, 48]. Transitioning to living alone might strip them of this identity. The loss of a “care-recipient” may weaken their motivation for self-care, which has long been sustained by a sense of familial responsibility [39]. Moreover, the shift from being a caregiver to a care-seeker may involve psychological friction that further inhibits help-seeking behavior. This identity-based mechanism offers a plausible explanation for why older women experience a more significant decline in healthcare utilization when living alone.

### Limitations and future research

This study has several limitations. First, although we employed a longitudinal design capable of controlling for time-invariant individual heterogeneity, the risk of selection bias cannot be entirely eliminated. For instance, individuals with certain unobserved traits (such as higher pain tolerance or inherent psychological resilience) may self-select into living alone. While our fixed-effects models accounted for time-invariant unobserved variables, they remain unable to fully capture time-varying unobserved factors—such as sudden deteriorations in health status or specific household dynamics—which may simultaneously influence individuals’ residential decisions and healthcare-seeking behaviors.

Second, regarding variable measurement, our assessment of primary care preferences relied on self-reported usual choices rather than directly observed medical records. The conceptual distinction between such “stated preferences” and “revealed behavior” is critical: our findings reflect a tendency of preference under specific constraints, rather than realized patterns of healthcare utilization.

Furthermore, due to data limitations, healthcare-seeking behavior and primary care preferences were analyzed using two independent subsamples. This restricts the direct comparability between the two sets of results and hinders our ability to draw more integrated conclusions. Although we paid considerable attention to potential sample biases in the analysis, thereby strengthening the internal validity of each finding, this data constraint remains a factor to be considered when interpreting the study’s conclusions.

Future research could be enhanced in the following aspects: incorporating direct psychological assessment tools and combining survey data with objective medical records to more comprehensively uncover underlying mechanisms; utilizing unified datasets that integrate multiple types of healthcare behaviors to improve comparability across outcomes; and further investigating the influence of different types of household structures—such as multigenerational households or shared living arrangements—on healthcare access and preferences, thereby providing a more nuanced understanding.

## Conclusion

In summary, this study demonstrates that living alone significantly suppresses healthcare-seeking behavior among Chinese older adults, while also redirecting their medical preferences toward primary care institutions. This dual pattern reflects not merely individual choice, but rather a consequential adaptation shaped by institutional constraints, socioeconomic disparities, and gendered vulnerabilities. Heterogeneity analysis further reveals that financial resources cannot compensate for the absence of social support, underscoring the non-substitutable role of family in facilitating healthcare navigation. Moreover, identity theory helps to explain the more pronounced reduction in care-seeking among older women, as the loss of the caregiver role disrupts established self-care motivations.

To address these challenges, a integrated policy approach is recommended. On the demand side, community-based support systems—including proactive health monitoring, accompaniment services, and tailored health outreach—should be strengthened to functionally substitute for missing family support. On the supply side, policymakers should prioritize enhancing the clinical capacity and referral efficiency of primary care institutions, which is crucial for building public trust and establishing them as credible first-contact providers. We need to place special emphasis on developing gender-sensitive interventions that effectively address the distinct psychosocial vulnerabilities of older women living alone. By establishing a more responsive and trustworthy primary care system supported by robust community networks, China can effectively prevent the growing prevalence of solitary living from leading to systematic health neglect among older adults.

## Supplementary Information

Supplementary Material 1.

## Data Availability

The study used data freely available at https://cfpsdata.pku.edu.cn/#/home.

## Abbreviations

CFPS: China Family Panel Studies
PHC: Primary health care
CLHLS: Chinese Longitudinal Healthy Longevity Survey
ISSS: Institute of Social Science Survey
URBMI: Urban Resident Basic Medical Insurance

## Footnotes

1 Our analytical sample was restricted to observations with complete data on all key variables, including living arrangements, healthcare utilization, and baseline controls.

2 In Supplementary Table S2, we report the distribution of older adults living alone and not living alone across each variable, as a proportion of the total sample.
