# Supplementary material for: Does living alone reshape healthcare use? Longitudinal evidence from older adults in China
Source: BMC Prim Care. 2026 Jan 7;27:38. doi: 10.1186/s12875-025-03164-7 (PMC12870296; doi:10.1186/s12875-025-03164-7)
Supplement: Supplementary file 1 — Supplementary Material 1. [file 12875_2025_3164_MOESM1_ESM.docx]

**Does Living Alone Reshape Healthcare Use? Longitudinal Evidence from Older Adults in China**

**Supplementary material**

**Supplementary Table S1** Definition of related variables.

**Supplementary Table S1** Definition of related variables.

| Variable Type | Variable Name | CFPS Questions | Variable Definition |
| --- | --- | --- | --- |
| Dependent variable | Living alone | Father: Co-residing or not  1-Yes  0-No  Mother: Co-residing or not  1-Yes  0-No  Spouse: Co-residing or not  1-Yes  0-No  Child 1: Co-residing or not  1-Yes  0-No  Child 2: Co-residing or not  1-Yes  0-No  Child 3: Co-residing or not  1-Yes  0-No  Child 4: Co-residing or not  1-Yes  0-No  Child 5: Co-residing or not  1-Yes  0-No  Child 6: Co-residing or not  1-Yes  0-No  Child 7: Co-residing or not  1-Yes  0-No  Child 8: Co-residing or not  1-Yes  0-No  Child 9: Co-residing or not  1-Yes  0-No  Child 10: Co-residing or not  1-Yes  0-No | If the respondent answers "No" to all of the above 13 questions, the binary variable for living arrangement equals 1, indicating living alone. If the respondent answers "Yes" to any of the 13 questions, the variable living arrangement equals 0, indicating co-residence with family members. |
| Independent variable | Consulted Doctors | In the past two weeks, have you experienced any physical discomfort?  1-Yes  0-No  For any illnesses experienced in the past two weeks, did you consult a doctor?  1-Yes  0-No | In the CFPS survey, respondents are initially asked: *"In the past two weeks, have you experienced any physical discomfort?"* If the respondent's answer is "No," they are not asked any further related questions. In other words, respondents who answer "No" cannot provide valid information regarding consulting a doctor. If the respondent's answer is "Yes," they are then asked a follow-up question: *"For any illnesses experienced in the past two weeks, did you consult a doctor?"* If the respondent answers "Yes" to this question, the binary variable *Consulted Doctors* is assigned a value of 1; otherwise, it is assigned a value of 0. |
|  | Primary Health Care Preference | If you consult a doctor, where do you typically go?  1. General hospital  2. Specialized hospital  3. Community health service center/township health center  4. Community health service station/village clinic  5. Private clinic  Note: 1. "General hospitals" refer to large hospitals that typically provide diagnosis and treatment for a wide range of diseases.  2. "Specialized hospitals" refer to hospitals focused on specific types of diseases, such as maternity hospitals.  3. "Community health service centers/township health centers" refer to medical institutions located at the street or township level, primarily for diagnosing and treating common illnesses.  4. "Community health service stations/village clinics" refer to medical institutions located at the residential committee or village level, primarily for diagnosing and treating common illnesses.  5. "Clinics" generally refer to private clinics or village/community clinics with basic medical facilities. | Primary Health Care Preference equals to 1 if the respondent answers 3, 4 and 5. Primary Health Care Preference equals to 0 if the respondent answers 1 and 2.  Note: Non-PHC institutions (including general hospitals and speciality hospitals) and PHC institutions (including community healthcare centres/township hospitals, community healthcare stations/village clinics, and clinics). |
| Control variables | Age | CFPS automatically generates age | The age of respondent |
|  | Age square | CFPS automatically generates age | The age square of respondent  Age square = Age * age |
|  | Married | What is your marital status?  1. Single (never married)  2. Married (currently has a spouse)  3. Cohabiting  4. Divorced  5. Widowed | The Marital status of the respondent.  1. Single (never married)  2. Married (currently has a spouse)  3. Cohabiting  4. Divorced  5. Widowed  Categorical variable (with "single/never married" as the reference group) |
|  | Chronic illness | In the past six months, have you been diagnosed by a doctor with a chronic disease?  1-Yes  0-No | In the past six months, whether the respondent been diagnosed by a doctor with a chronic disease?  1-Yes  0-No |
|  | Type of insurance | Which types of pension insurance programs are you enrolled in?  1. Receiving a pension after retirement from a government agency or public institution  2. Basic pension insurance  3. Enterprise supplementary pension insurance  4. Commercial pension insurance  5. Rural pension insurance (Old Farmers' Pension Scheme)  6. New Rural Social Pension Insurance (New Rural Pension Scheme)  7. Urban Resident Pension Insurance  8. None of the above | Which types of pension insurance programs are the respondents enrolled in?  1-None insurance (answers 8)  2-Public medical care (answers 4)  3-Urban employee medical insurance (answers 1,2 &3)  4-Urban resident medical insurance (answers 7)  5-New rural cooperative medical care (answers 5 & 6)  Categorical variable (with "None insurance" as the reference group) |
|  | Intensity of physical | How would you rate the severity of the illness or injury you experienced in the past two weeks?  1. Not serious  2. Moderate  3. Severe | How would the respondent rate the severity of the illness or injury he/she experienced in the past two weeks?  1. Not serious  2. Moderate  3. Severe  Categorical variable (with "Not serious" as the reference group) |
|  | Household income | Total household income in the last year including labour and non-labour income (from CFPS original data set) | Total household income in the last year including labour and non-labour income |
|  | Independent outdoor activity | Are you able to independently go to outdoor activities (walk about 300 meters, such as go to station, shopping center, parking lot)? | Binary variable (if the respondent's answer is "Yes", then independent outdoor activity equals 1, if the respondent's answer is "No", then independent outdoor activity equals 0). |
|  | Household size | Total Number of Household Members (from CFPS original data set) | Total Number of Household Members (from CFPS original data set) |
| Heterogeneous variables | Gender | Gender of the respondents   1. Male 2. Female | Gender of the respondents  0-Femal  1-Male |
|  | Hukou | Type of respondent’s Hukou  1.Rural Hukou  3. Non-rural Hukou  5. No Hukou  7.Urban Hukou | Respondent’s Hukou Type   1. Rural Hukou 2. Urban Hukou |
|  | Household income | Total household income in the last year including labour and non-labour income (from CFPS original data set) | Total household income in the last year including labour and non-labour income  0 - low income (below average)  1 - high income (above average) |

**Supplementary Table S2** Descriptive statistics

| **Variables** | **Whether consulted a doctor** | | | | | | | |
| --- | --- | --- | --- | --- | --- | --- | --- | --- |
|  | **All observations** | | | **Not living alone** | | **Living alone** | | **MeanDiff** |
|  | **N** | **Mean** | **sd** | **N** | **Mean** | **N** | **Mean** |  |
| **Age** | 3911 | 71.35 | 9.97 | 3035 | 70.63 | 876 | 73.85 | -3.22^***^ |
| **Household income** | 3911 | 34554.93 | 45287.53 | 3035 | 39140.29 | 876 | 18668.45 | 20471.85^***^ |
| **Household size** | 3911 | 4.72 | 2.23 | 3035 | 5.52 | 876 | 1.92 | 3.60^***^ |
|  | **Freq.** | **Percent** | **Cum.** | **Freq.** | **Percent** | **Freq.** | **Percent** |  |
| **Whether consulted a doctor** |  |  |  |  |  |  |  |  |
| No | 716 | 18.31 | 18.31 | 556 | 77.65 | 160 | 22.35 | 0.00 |
| Yes | 3,195 | 81.69 | 100 | 2,479 | 77.59 | 716 | 22.41 |  |
| **Living alone** |  |  |  |  |  |  |  |  |
| No | 3,035 | 77.6 | 77.6 |  |  |  |  |  |
| Yes | 876 | 22.4 | 100 |  |  |  |  |  |
| **Marital status** |  |  |  |  |  |  |  |  |
| Single | 113 | 3.00 | 2.89 | 12 | 10.62 | 101 | 89.38 | -2.23^***^ |
| Married (with spouse) | 2,887 | 73.82 | 76.71 | 2,823 | 97.78 | 64 | 2.22 |  |
| Cohabiting | 54 | 1.38 | 78.09 | 53 | 98.15 | 1 | 1.85 |  |
| Divorced | 86 | 2.20 | 80.29 | 9 | 10.47 | 77 | 89.53 |  |
| Widowed | 771 | 19.71 | 100 | 138 | 17.90 | 633 | 82.10 |  |
| **Chronic illness** |  |  |  |  |  |  |  |  |
| No | 2,202 | 56.3 | 56.3 | 1,710 | 77.66 | 492 | 22.34 | 0.00 |
| Yes | 1,709 | 43.7 | 100 | 1,325 | 77.53 | 384 | 22.47 |  |
| **Type of insurance** |  |  |  |  |  |  |  |  |
| None insurance | 321 | 8.21 | 8.21 | 232 | 72.27 | 89 | 27.73 | 0.17^***^ |
| Public medical care | 120 | 3.07 | 11.28 | 83 | 69.17 | 37 | 30.83 |  |
| Urban employee medical insurance | 386 | 9.87 | 21.15 | 299 | 77.46 | 87 | 22.54 |  |
| Urban resident medical insurance | 291 | 7.44 | 28.59 | 209 | 71.82 | 82 | 28.18 |  |
| New rural cooperative medical care | 2,793 | 71 | 100 | 2,212 | 79.20 | 581 | 20.80 |  |
| **Intensity of physical discomfort** |  |  |  |  |  |  |  |  |
| Not serious | 534 | 13.65 | 13.65 | 430 | 80.52 | 104 | 19.48 | -0.05 |
| Moderate | 1,404 | 35.9 | 49.55 | 1,077 | 76.71 | 327 | 23.29 |  |
| Serious | 1,973 | 50.45 | 100 | 1,528 | 77.45 | 445 | 22.55 |  |
| **Gender** |  |  |  |  |  |  |  |  |
| Female | 1861 | 57.19 | 57.19 | 1,367 | 73.36 | 494 | 26.54 | 0.09*** |
| Male | 1393 | 42.81 | 100.00 | 1112 | 79.83 | 281 | 20.17 |  |
| **Hukou** |  |  |  |  |  |  |  |  |
| Rural | 2882 | 77.81 | 77.81 | 2250 | 78.07 | 632 | 21.93 | -0.28^***^ |
| Urban | 822 | 22.19 | 100 | 605 | 72.72 | 227 | 27.28 |  |
| **Independent outdoor activity** |  |  |  |  |  |  |  |  |
| No | 407 | 11.06 | 11.06 | 309 | 75.92 | 98 | 24.08 | 0.01 |
| Yes | 3,273 | 88.94 | 100.00 | 2,546 | 77.79 | 727 | 22.21 |  |
| **Total** | 3,911 | 100 |  | 3,035 | 77.60 | 876 | 22.40 |  |
| **Variables** | **Primary Health Care** | | | | | | | |
|  | **All observations** | | | **Not living alone** | | **Living alone** | | **MeanDiff** |
|  | **N** | **Mean** | **sd** | **N** | **Mean** | **N** | **Mean** |  |
| **Age** | 11956 | 71.34 | 10.12 | 9775 | 70.76 | 2181 | 73.93 | -3.17^***^ |
| **Household income** | 11956 | 40121.52 | 50144.35 | 9775 | 44672.23 | 2181 | 19725.74 | 24946.49^***^ |
| **Household size** | 11956 | 4.83 | 2.21 | 9775 | 5.49 | 2181.00 | 1.89 | 2.4^***^ |
|  | **Freq.** | **Percent** | **Cum.** | **Freq.** | **Percent** | **Freq.** | **Percent** |  |
| **PHC** |  |  |  |  |  |  |  |  |
| No | 4,207 | 35.19 | 35.19 | 3,471 | 82.51 | 736 | 17.49 | -2.00 |
| Yes | 7,749 | 64.81 | 100 | 6,304 | 81.35 | 1,445 | 18.65 |  |
| **Living alone** |  |  |  |  |  |  |  |  |
| No | 9,775 | 81.76 | 81.76 |  |  |  |  |  |
| Yes | 2,181 | 18.24 | 100 |  |  |  |  |  |
| **Marital status** |  |  |  |  |  |  |  |  |
| Single | 300 | 2.51 | 2.51 | 68 | 22.67 | 232 | 77.33 | -2.18^***^ |
| Married (with spouse) | 9,466 | 79.17 | 81.68 | 9,242 | 97.63 | 224 | 2.37 |  |
| Cohabiting | 19 | 0.16 | 81.84 | 15 | 78.95 | 4 | 21.05 |  |
| Divorced | 293 | 2.45 | 84.29 | 121 | 41.30 | 172 | 58.70 |  |
| Widowed | 1,878 | 15.71 | 100 | 329 | 17.52 | 1,549 | 82.25 |  |
| **Chronic illness** |  |  |  |  |  |  |  |  |
| No | 8,546 | 71.48 | 71.48 | 7,066 | 82.68 | 1,480 | 17.32 | -0.04^***^ |
| Yes | 3,410 | 28.52 | 100 | 2,709 | 79.44 | 701 | 20.56 |  |
| **Type of insurance** |  |  |  |  |  |  |  |  |
| None insurance | 955 | 7.99 | 7.99 | 740 | 77.49 | 215 | 22.51 | 0.06^**^ |
| Public medical care | 430 | 3.6 | 11.58 | 353 | 82.09 | 77 | 17.91 |  |
| Urban employee medical insurance | 1,451 | 12.14 | 23.72 | 1,204 | 82.98 | 247 | 17.02 |  |
| Urban resident medical insurance | 1,044 | 8.73 | 32.45 | 867 | 83.05 | 177 | 16.95 |  |
| New rural cooperative medical care | 8,076 | 67.55 | 100 | 6,611 | 81.86 | 1,465 | 18.14 |  |
| **Gender** |  |  |  |  |  |  |  |  |
| Female | 4776 | 48.82 | 48.82 | 3728 | 78.06 | 1048 | 21.94 | 0.09^***^ |
| Male | 5007 | 51.18 | 100 | 4,172 | 83.32 | 835 | 16.68 |  |
| **Hukou** |  |  |  |  |  |  |  |  |
| Rural | 8,258 | 73.05 | 73.05 | 6701 | 81.15 | 1577 | 18.85 | -0.02 |
| Urban | 3047 | 26.95 | 100 | 2494 | 81.85 | 553 | 18.15 |  |
| **Independent outdoor activity** |  |  |  |  |  |  |  |  |
| No | 725 | 6.39 | 6.39 | 544 | 75.03 | 181 | 24.97 | 0.03^***^ |
| Yes | 10,615 | 93.61 | 100.00 | 8740 | 82.34 | 1875 | 17.66 |  |
| **Total** | 11956 | 100 |  | 9775 | 81.76 | 2181 | 18.24 |  |
| *Note: A slight reduction in observations occurred in the robustness checks and heterogeneity analysis due to missing values in key variables. In the "Whether consulted a doctor" panel (baseline N=3,911), complete cases numbered 3,254 for gender (-16.8%), 3,704 for hukou (-5.3%), and 3,680 for independent outdoor activity (-5.9%). Similarly, in the "Primary Health Care" panel (baseline N=11,956), complete cases numbered 9,783 for gender (-18.2%), 11,305 for hukou (-5.4%), and 11,304 for independent outdoor activity (-5.5%).* | | | | | | | | |

**Supplementary Table S3.1** Robustness analysis (Consulted a doctor).

|  | (1) | (2) | (3) | (4) | (5) | (6) |
| --- | --- | --- | --- | --- | --- | --- |
|  | Conditional fixed-effects model | Population-averaged model | Control Independent outdoor activity | Control Household size | Clustered at individual level | Aged 65 and above |
| **Living alone** | -0.760 | -0.100^**^ | -0.208^**^ | -0.207^**^ | -0.202^***^ | -0.423^**^ |
|  | (1138.752) | (0.046) | (0.085) | (0.077) | (0.058) | (0.171) |
| **Age** | -0.002 | -0.011 | -0.012 | -0.010 | -0.012 | 0.020 |
|  | (12.231) | (0.007) | (0.014) | (0.011) | (0.012) | (0.029) |
| **Age square** | 0.000 | 0.000 | 0.000 | 0.000 | 0.000 | -0.000 |
|  | (0.080) | (0.000) | (0.000) | (0.000) | (0.000) | (0.000) |
| **Married** | -0.018 | -0.097^**^ | -0.119 | -0.187 | -0.190 | -0.034 |
|  | (0.692) | (0.039) | (0.341) | (0.329) | (0.231) | (0.601) |
| **Cohabiting** | 0.069 | -0.036 | -0.055 | -0.131 | -0.139 | -0.203 |
|  | (5.1e+05) | (0.112) | (0.419) | (0.392) | (0.285) | (0.534) |
| **Divorced** | 0.270 | -0.020 | 0.349 | 0.204 | 0.205 | 0.627 |
|  | (0.233) | (0.039) | (0.342) | (0.241) | (0.177) | (0.515) |
| **Widowed** | 0.253 | -0.006 | 0.095 | 0.003 | 0.002 | 0.330 |
|  | (0.279) | (0.027) | (0.340) | (0.306) | (0.213) | (0.547) |
| **Chronic illness** | 0.059 | 0.111^***^ | 0.063^***^ | 0.068^***^ | 0.068^***^ | 0.074^***^ |
|  | (726.724) | (0.012) | (0.016) | (0.015) | (0.016) | (0.024) |
| **Household income** | 0.010 | 0.009^*^ | 0.013 | 0.011 | 0.011 | 0.013 |
|  | (80.910) | (0.005) | (0.009) | (0.009) | (0.007) | (0.012) |
| **Public medical care** | 0.125 | 0.066 | 0.121 | 0.117 | 0.113 | -0.009 |
| **Urban employee medical insurance** | (1544.868) | (0.040) | (0.079) | (0.077) | (0.069) | (0.087) |
| **Urban resident medical insurance** | 0.065 | -0.025 | 0.038 | 0.023 | 0.020 | -0.052 |
| **New rural cooperative medical care** | (329.630) | (0.032) | (0.069) | (0.063) | (0.051) | (0.084) |
| **Public medical care** | 0.078 | 0.031 | 0.109^*^ | 0.080 | 0.079^*^ | 0.057 |
| **Urban employee medical insurance** | (451.519) | (0.031) | (0.054) | (0.050) | (0.042) | (0.096) |
| **Urban resident medical insurance** | 0.050 | 0.051^**^ | 0.050 | 0.033 | 0.030 | 0.012 |
|  | (221.804) | (0.023) | (0.037) | (0.030) | (0.031) | (0.056) |
| **Moderate physical discomfort** | 0.140 | 0.126^***^ | 0.151^***^ | 0.149^***^ | 0.148^***^ | 0.129^*^ |
|  | (430.281) | (0.021) | (0.040) | (0.036) | (0.026) | (0.065) |
| **Serious physical discomfort** | 0.194 | 0.212^***^ | 0.238^***^ | 0.235^***^ | 0.235^***^ | 0.222^***^ |
|  | (1156.684) | (0.021) | (0.040) | (0.037) | (0.027) | (0.058) |
| **Independent outdoor activity** |  |  | 0.001 |  |  |  |
|  |  |  | (0.024) |  |  |  |
| **Household size** |  |  |  | -0.012 |  |  |
|  |  |  |  | (0.010) |  |  |
| **Constant** |  |  | 1.048 | 1.084 | 1.157^**^ | -0.311 |
|  |  |  | (0.872) | (0.697) | (0.568) | (1.684) |
| **Number of observations** | 1234 | 3911 | 3571 | 3911 | 3911 | 2283 |
| **Adjusted R2** |  |  | 0.568 | 0.558 | 0.556 | 0.565 |
| * p < 0.1, ** p < 0.05, *** p < 0.01  Note: Columns (1)-(6) all employment-time and individual fixed effects. | | | | | | |

**Supplementary Table S3.2** Robustness analysis (Primary Health Care).

|  | (1) | (2) | (3) | (5) | (6) | (7) |
| --- | --- | --- | --- | --- | --- | --- |
|  | Conditional fixed-effects model | Population-averaged model | Control Independent outdoor activity | Control Household size | Clustered at individual level | Aged 65 and above |
| **Living alone** | 0.037 | 0.076^**^ | 0.102^*^ | 0.114^*^ | 0.109^**^ | 0.146^**^ |
|  | (0.273) | (0.030) | (0.054) | (0.060) | (0.046) | (0.065) |
| **Age** | -0.003 | -0.005 | -0.005 | -0.005 | -0.006 | -0.003 |
|  | (0.019) | (0.005) | (0.011) | (0.011) | (0.007) | (0.015) |
| **Age square** | 0.000 | 0.000 | 0.000 | 0.000 | 0.000 | 0.000 |
|  | (0.000) | (0.000) | (0.000) | (0.000) | (0.000) | (0.000) |
| **Married** | 0.044 | 0.005 | 0.249^***^ | 0.215^*^ | 0.213^**^ | 0.482^***^ |
|  | (0.486) | (0.048) | (0.080) | (0.109) | (0.101) | (0.143) |
| **Cohabiting** | 0.102 | 0.002 | 0.362^***^ | 0.347^**^ | 0.333^**^ | 0.421^**^ |
|  | (1.246) | (0.103) | (0.119) | (0.147) | (0.132) | (0.159) |
| **Divorced** | 0.011 | -0.137^***^ | 0.115 | 0.108 | 0.109 | 0.172 |
|  | (0.178) | (0.051) | (0.079) | (0.090) | (0.092) | (0.105) |
| **Widowed** | 0.013 | -0.059 | 0.152 | 0.091 | 0.094 | 0.289^**^ |
|  | (0.243) | (0.040) | (0.096) | (0.124) | (0.101) | (0.118) |
| **Chronic illness** | -0.017 | -0.083^***^ | -0.061^***^ | -0.061^***^ | -0.062^***^ | -0.054^***^ |
|  | (0.129) | (0.009) | (0.015) | (0.013) | (0.011) | (0.016) |
| **Household income** | -0.001 | -0.011^***^ | -0.004 | -0.003 | -0.002 | -0.011 |
|  | (0.004) | (0.004) | (0.007) | (0.007) | (0.004) | (0.008) |
| **Public medical care** | -0.020 | -0.258^***^ | -0.088^*^ | -0.088^*^ | -0.090^**^ | -0.067 |
| **Urban employee medical insurance** | (0.154) | (0.027) | (0.048) | (0.049) | (0.035) | (0.062) |
| **Urban resident medical insurance** | -0.013 | -0.213^***^ | -0.061 | -0.065^*^ | -0.066^**^ | -0.073^*^ |
| **New rural cooperative medical care** | (0.099) | (0.021) | (0.037) | (0.037) | (0.029) | (0.040) |
| **Public medical care** | -0.007 | -0.138^***^ | -0.032 | -0.036 | -0.036 | -0.050 |
| **Urban employee medical insurance** | (0.051) | (0.022) | (0.032) | (0.031) | (0.027) | (0.041) |
| **Urban resident medical insurance** | 0.006 | 0.113^***^ | 0.024 | 0.020 | 0.021 | 0.002 |
|  | (0.050) | (0.016) | (0.029) | (0.029) | (0.019) | (0.033) |
| **Independent outdoor activity** |  |  | 0.030 |  |  |  |
|  |  |  | (0.030) |  |  |  |
| **Household size** |  |  |  | 0.003 |  |  |
|  |  |  |  | (0.007) |  |  |
| **Constant** |  |  | 0.647 | 0.715 | 0.742^**^ | 0.503 |
|  |  |  | (0.485) | (0.476) | (0.315) | (0.761) |
| **Number of observations** | 5281 | 11956 | 11119 | 11956 | 11956 | 6973 |
| **Adjusted R2** |  |  | 0.594 | 0.589 | 0.588 | 0.613 |
| * p < 0.1, ** p < 0.05, *** p < 0.01  Note: Columns (1)-(6) all employment-time and individual fixed effects. | | | | | | |
